# Consistency and Discrepancy between Visibility and PM_2.5_ Measurements: Potential Application of Visibility Observation to Air Quality Study

**DOI:** 10.3390/s23020898

**Published:** 2023-01-12

**Authors:** Ye Fei, Jie Liao, Zhisen Zhang

**Affiliations:** National Meteorological Information Center (NMIC), China Meteorological Administration (CMA), Beijing 100081, China

**Keywords:** PM_2.5_, visibility, surface extinction coefficient, abrupt changes, dataset

## Abstract

High-quality measurements of air quality are the highest priority for understanding widespread air pollution. Visibility has been widely suggested to be a good alternative to PM_2.5_ concentration as a measure. In this study, the similarities and differences between visibility and PM_2.5_ measurements in China are checked and the results reveal the potential application of visibility observation to the study of air quality. Based on the quality-controlled PM_2.5_ and visibility data from 2016 to 2018, the nonparametric Spearman correlation coefficient (*ρ*) values between stations for PM_2.5_ and visibility-derived surface extinction coefficient (b_ext_) decrease as the station distance (*R*) increases. Some relatively low *ρ* values (<0.4) occur in regions characterized by the lowest (background) levels of PM_2.5_ and b_ext_ values, for example, the Tibetan and Yungui Plateau. The relatively lower *ρ* for b_ext_ compared to PM_2.5_ is probably caused by the predefined maximum threshold of visibility measurements (generally 30 km). A significant correlation between PM_2.5_ and b_ext_ is derived in most stations and relatively larger ρ values are evident in eastern China (Northeast China excluded) and in winter (the national median *ρ* is 0.67). The abrupt changes in specific mass extinction efficiency (α_ext_) imply a potentially large influence of alternation of visibility sensors or recalibrations on visibility measurements. The b_ext_ data are thereafter corrected by comparison to the reference measurements at the adjacent stations, which leads to a three-year quality assured of visibility and b_ext_ datasets.

## 1. Introduction

As a byproduct of rapid and energy-intensive economic development, a huge amount of precursors and particles are emitted into the atmosphere. Anthropogenic emissions, working with natural emissions such as dust and biomass burning, lead to a thick layer of extensive haze covering thousands or tens of thousands of acres. Large areas of haze occur all over the world but are more often observed in developing countries. For example, a dense blanket of polluted air often occurs in eastern China, especially over the North China Plain in winter and in central eastern China in the crop harvest season [1,2,3,4,5]. Similarly, a thick brownish-gray haze often covers much of the Ganges Plain in the pre-monsoon season [6].

Measurements are the highest priority for understanding the formation and maintenance of widespread haze events and their extensive impacts. Ground measurement is the usual approach to obtaining accurate measurements of air quality indexes, among which PM_2.5_ concentration (particulate matter with aerodynamic diameter < 2.5 μm) is the key component. PM_2.5_ fine particles are critical because of their extensive impacts. PM_2.5_ is the major factor leading to a reduction in visibility in the absence of precipitation. Visibility has reduced in the past half-century in many regions of the world owing to the increase in aerosol emissions into the atmosphere [7,8,9]. An increase in PM_2.5_ also lead to the reduction in solar energy reaching the ground across the world from 1960 to the 1980s that was known as global dimming [10,11,12]. As cloud condensation nuclei, cloud formation and lifetime are intimately linked to fine particles in the atmosphere, which is the hottest debated issue in the study of climate change [13,14]. Moreover, PM_2.5_ can penetrate deeply into the lungs of human beings and thereby lead to disastrous impacts on human health [15,16].

Real-time hourly PM_2.5_ concentration measurements are available in many countries, which has greatly contributed to the study of air quality and related topics. The data are widely used in the following ways. (1) to study how and why air quality varies spatiotemporally [17,18]; (2) to validate model and satellite remote sensing products [19,20]; (3) to establish aerosol hygroscopic growth parameterization [21,22]; and (4) to study effects of air quality on human health [23].

Because air quality stations in mainland China are mainly located in urban areas and the spatial variability of air quality is substantial, we need extra measures to enhance the spatial coverage of national air quality networks. Local governments have established provincial air quality networks, which can, to some extent, fill the gap, but the stations are still mostly located in urban areas. Satellite imaging is widely suggested to be a potential alternative for monitoring large-scale air quality. Much effort has been paid and great progress has been made in this approach [24,25,26]. However, ground measurements are required by satellite remote sensing algorithms to improve their performance. Moreover, the temporal coverage of satellite retrieval is highly sensitive to the occurrence of clouds, snow, and even heavy aerosol events, all of which make image retrieval impossible [20].

Meteorological visibility is one of the operational meteorological observation parameters that reflect the abundance of solid and liquid particles in the atmosphere. The reduction in visibility is likely due to fogs or enhancements of natural and/or anthropogenic aerosols [27,28]. It is widely used as a good proxy for aerosols in the absence of rain and fog [7,29,30]. Traditionally, visibility (with units of km) is measured manually every 1–8 h, following the general rules outlined by the World Meteorological Organization. Human observation is subjective in nature, and may, therefore, eventually produce notable uncertainties. More importantly, in order to reduce cost, human observation has been gradually replaced by instrumental observation. For example, the China Meteorological Administration (CMA) established a national visibility network using a forward-scatter visibility sensor that provides hourly visibility measurements at more than 2000 automatic stations across the country [31]. This not only plays an important role in meteorological and air traffic services but is also a potential complementary network for the study of air quality. One would expect much more from this automatic observation because of the following reasons. First, automatic observation is objective in nature, which lends it good reproducibility and comparability. Second, it can provide hourly or even once-a-minute measurements. Third, automatic visibility observation can be made under harsh environments and therefore has larger spatial coverage. At the same time, these advantages are overwhelmingly dependent on good maintenance and the regular calibration of sensors.

Both measurements (PM_2.5_ and visibility) are not free of measurement uncertainties and in some cases, the uncertainties may exceed the specified expectation. Many methods are thereby developed to check the data quality of meteorological and environmental data [32]. These methods mainly rely on the spatiotemporal variability of a simple variable, but one should keep in mind that it is not easy to detect true outliers from spurious data with large measurement uncertainties. Because aerosol measurements are often associated with notable uncertainties, it is strongly recommended to compare as many measurements of the same quantities as possible by different measurement techniques. This is a robust tool to assess measurement uncertainties and detect outliers. PM_2.5_ measurements by the Tapered Element Oscillating Microbalance (TEOM) or the beta absorption method (BAM) represent dry particulate matter mass (RH < 40%). The visibility measured by the visibility sensor represents the ambient extinction in the atmosphere, which is related to PM_2.5_ concentrations when aerosol hygroscopic growth is carefully considered. A good linear relation between PM_2.5_ and the visibility-derived extinction coefficient (b_ext_) is observed based on short-term measurements [33,34]. However, analysis of the consistency and discrepancy between PM_2.5_ and visibility measurements may show a few important issues which have not been revealed by previous univariate analyses of measurement uncertainties. This tells us that caution should be taken in applying visibility measurements in the study of air quality. The rest of this paper is structured as follows. Section 2 introduces the data and method. Section 3 presents major results; and the discussion and conclusions are summarized in Section 4.

## 2. Data and Method

**Figure 1:**
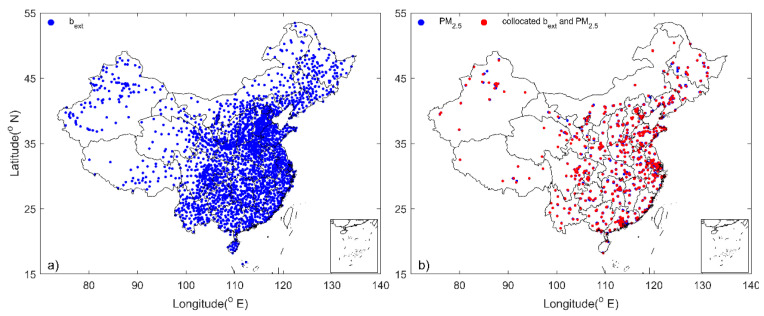
The spatial distribution of the visibility (b_ext_) stations (**a**), PM_2.5_ stations (blue dots in (**b**)), and collocated b_ext_ and PM_2.5_ stations (red dots in (**b**)).

Hourly PM_2.5_ and PM_10_ concentrations (μg m^−3^) are available at ~1600 stations that cover at least one-year worth of measurements for the period from 2016 to 2018. The spatial distribution of the stations is shown in Figure 1, which clearly shows that the stations are clustered in urban regions, for example, 12 national stations are mainly located in urban and suburban areas of Beijing. Conversely, the visibility stations are much more evenly distributed, especially in eastern China (Figure 1). Hourly visibility data (km) are available from the National Meteorological Information Center (NMIC) of CMA at 2395 stations for the period from 2016 to 2018. Visibility is divided by 2.996 to derive the extinction coefficient (b_ext_: Mm^−1^) according to the instrument manual [35]. Hourly relative humidity (RH) measurements are also available from the NMIC to allow a humidity correction of b_ext_.

The data quality assurance procedure of Wu et al. [36] is adopted in this paper to provide quality control for the PM_2.5_ and b_ext_ data. In general, measurements that are greatly deviated from the observations at the adjacent time or in neighboring areas or have a very low temporal variance are classified as outliers. Regular calibration of instruments and consistency between PM_2.5_ and PM_10_ are checked for assurance of PM_2.5_ data. For b_ext_ data, the consistency between 10 min and 1 min measurements is checked for assurance [31].

In order to check the relationship between PM_2.5_ and b_ext_, PM_2.5_ measurements at stations no further than 25 km from a visibility station are averaged to match b_ext_ data. This collocation procedure trades off two requirements. One is to match PM_2.5_ and b_ext_ at stations that are adjacent to each other as close as possible in order to minimize the potential effect of spatial variation; the other is to collocate substantially more b_ext_ and PM_2.5_ stations for statistical analysis. Simultaneous measurements of PM_2.5_ and b_ext_ are available at 502 stations (Figure 1b) that are used for the following analysis.

Spatial Spearman correlation coefficients (*ρ*) of PM_2.5_ concentration and ambient b_ext_ between stations are calculated separately. The variation of *ρ* with the distance between stations (*R*) is studied. A few abnormally lower *ρ* values are found, especially for b_ext_ data in some provinces, which implies potentially larger random errors of visibility and then b_ext_ measurements than that of PM_2.5_.

The linear regression between hourly PM_2.5_ and b_ext_ for RH < 40% are performed to check their consistency, the slope of which represents the specific mass extinction efficiency (α_ext_). α_ext_ can also be calculated directly by dividing b_ext_ by PM_2.5_. In some stations, α_ext_ shows a few abrupt changes that are detected by a simple but effective approach developed by Killick et al. [37]. This implies a potentially large influence of alternation of visibility sensors or re-calibrations. The visibility or b_ext_ data of the collocated 502 stations are detected and corrected by comparison to the reference PM_2.5_ measurements, and thereby the visibility in these stations is set as the benchmark. The detection and correction of the remaining 1893 visibility stations should compare to the visibility measures in nearby stations. When there is a benchmark station nearby (<30 km), the visibility measurement of the benchmark station is set as the baseline. Otherwise, the regional average visibility of all meteorological stations within 100 km is set as the reference series. After all corrections are completed, a dataset consisting of three years worth of visibility and b_ext_ data is finally produced.

## 3. Results

**Figure 2:**
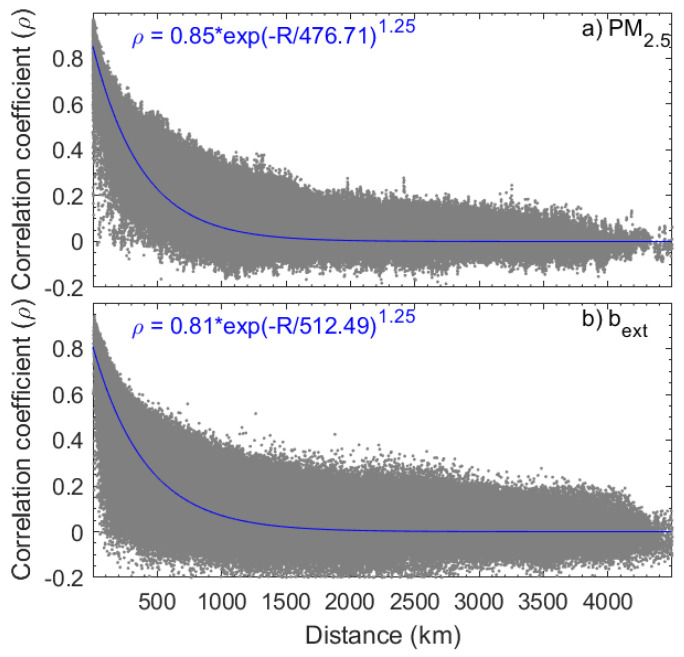
The scatterplot of correlation coefficient (*ρ*) versus station distance (*R*) for PM_2.5_ (**a**) and b_ext_ (**b**). An exponential equation is derived to describe the relationship between *ρ* and *R*, which is also shown.

**Figure 3:**
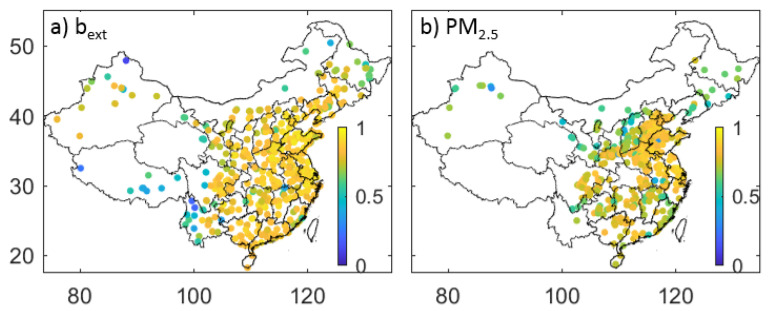
Spearman correlation coefficient (*ρ*) between stations with a distance of less than 50 km for PM_2.5_ (**b**) and b_ext_ (**a**).

Figure 2 shows the scatterplot of *ρ* versus *R*. The *ρ* values between two stations for PM_2.5_ and b_ext_ are calculated because both quantities are not normally distributed. Not surprisingly, *ρ* decreases as *R* increases. The decay of *ρ* with *R* may be represented by an exponential equation [38].

(1) $$\rho =\rho_{0}\exp{\left(-\frac{R}{R_{0}}\right)}^{\gamma }$$

where the coefficient *R*_0_ indicates the distance at which *ρ* decreases by a factor of *e*, representing the horizontal scale of the correlation. *ρ*_0_, the zero intercept, represents *ρ* where the station distance is zero. The appropriate value for *γ*, the parameter determining how *ρ* decays with *R*, is not obvious. A range of possible values is considered that leads to substantially large variations of *R*_0_, but not *ρ*_0_. As suggested by Liu et al. [39], *ρ*_0_ provides information on the uncertainty of the measurements ($\sigma^{2}\left(e\right)$), i.e.,

(2) $$\sigma^{2}\left(e\right)=\left(1-\rho_{0}\right)\sigma^{2}\left(M\right)$$

where $\sigma^{2}\left(M\right)$ represents the variance of the measurements. The relative uncertainty of PM_2.5_ is 15% and b_ext_ is 20%, which are close to the expected measurement uncertainties. An interesting feature of Figure 2 is that some relatively lower *ρ* values (<0.4) are observed for *R* < 50 km, not only for PM_2.5_ but also for b_ext_. A further check of these low *ρ* values shows that they occur in regions characterized by the lowest (background) levels of PM_2.5_ and b_ext_ values, for example, in the Tibetan and Yungui Plateau. This can be clearly shown by Figure 3 in which mean *ρ* values of PM_2.5_ and b_ext_ between stations with *R* < 50 km are shown. Relative smaller *ρ* values are also found in Northeast China where PM_2.5_ and b_ext_ exceed that in the Tibetan and Yungui Plateau.

*ρ* for visibility and, therefore, b_ext_, between stations are smaller than that of PM_2.5_, which can be evident from Figure 2 and their fitting equations. The most likely explanation is that the maximum visibility is generally set to be 30 km (about 100 M m^−1^ for b_ext_). Given the fact that α_ext_ is about 5 m^2^ g^−1^ in eastern [33] and southwest China [34], the threshold of visibility prevents visibility measurements from resolving PM_2.5_ variation from a few to 20–30 μg m^−3^. In other words, visibility data cannot reflect a subtle variation in the background level of PM_2.5_ and b_ext_ as a result of their predefined maximum threshold. Therefore, it is recommended that raw b_ext_ data and visibility data be provided, which would be critical for the application of visibility data to the study of air quality study, or, more specifically, in the estimation of PM_2.5_ from visibility data.

**Figure 4:**
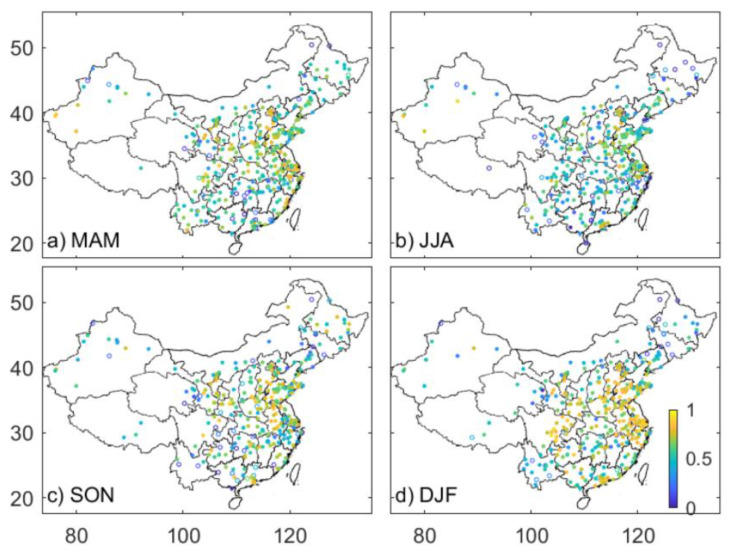
Spatial distribution of the Spearman correlation coefficient between PM_2.5_ and visibility-derived extinction coefficient (b_ext_). The solid (open) circle represents a significant correlation (at 99% of significance level) or not, respectively.

Figure 4 presents the seasonal spatial distribution of *ρ* between PM_2.5_ and b_ext_ under conditions with RH < 40%, under which temporal variation of PM_2.5_ should be expected to resemble that of b_ext_ because the hygroscopic growth is marginal. As summer (rainy season) match points between PM_2.5_ and b_ext_ are very limited if an RH of 40% is used in eastern China, we used an RH of 60% to produce sufficient match points to perform a robust statistical analysis. In order to minimize the potential effect of hygroscopic growth, the ambient light scattering enhancement (f_RH_) of PM_2.5_ is considered by using an empirical equation below.

(3) $$f_{\mathrm{RH}}=1+\kappa \cdot \frac{\mathrm{RH}}{\left(100-\mathrm{RH}\right)}$$

where κ is set to 0.096 according to the reference [29].

Consistent with our expectation, a significant correlation between PM_2.5_ and b_ext_ is derived in most stations. The percentages with significant correlation are 91%, 89%, 88%, and 93% from spring to winter. Relatively larger *ρ* values are evident in eastern China (Northeast China excluded), for example, in the Beijing-Tianjin-Hebei (BTH), the Yangtze Delta Region (YDR), and Pearl Delta Region (PDR). This is partly because of a wider range of PM_2.5_ and b_ext_ variation, which leads to a larger difference in ranks of the individual element and thereby *ρ*. In regions with low aerosol loading, for example, in the Tibetan autonomous region, Qinghai, and Yunnan Province, relatively smaller *ρ* values are observed. This is likely because the measurement uncertainties prevent the very subtle variation in both quantities from detection. Poor correlations are also evident at most stations in northeastern China, which seems not to be explained by the latter cause since PM_2.5_ and b_ext_ in this region generally exceed those in the Tibetan Plateau. Given the fact that visibility sensors are much more accurate for smaller visibility (<10–15 km) relative to larger visibility, this should be kept in mind when the visibility data are used to estimate PM_2.5_, especially in those regions dominated by PM_2.5_ concentration with tens of μg m^−3^.

Seasonally, relatively larger *ρ* values occur in winter (the national median *ρ* is 0.67) and smaller *ρ* values are observed in summer (the national median *ρ* is 0.54). This is likely because the variability in PM_2.5_ and b_ext_ in summer is smaller than that in winter. Furthermore, the temporal variation of aerosol chemical components, size distribution, and, therefore, the aerosol hygroscopic growth, may partly contribute to the smaller *ρ* values in summer, although the hygroscopic growth is considered in the analysis.

**Figure 5:**
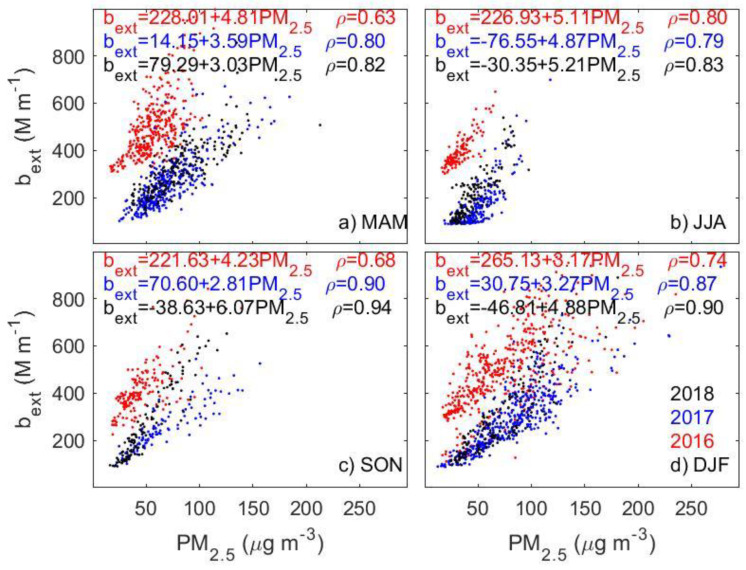
The scatter plot of seasonal matched points of PM_2.5_ and b_ext_ in different years at Yangzhou, Jiangsu province.

A temporal variation in the relationship between PM_2.5_ and b_ext_ is evident in some stations after a closer look at the scatter plot of PM_2.5_ and b_ext_ at each station. Figure 5 presents an example at Yangzhou, Jiangsu province, which shows a dramatically different relationship between PM_2.5_ and b_ext_ in 2016 relative to that in 2017 and 2018. This results in a poor correlation between the two quantities. The substantial change in the PM_2.5_-b_ext_ relationship may not be due to the temporal changes in aerosol compositions that mainly affects the slope of the regression analysis (i.e., α_ext_). We can see that a dramatic change in the intercept (from ~ −47 to ~265 M m^−1^ in winter) is observed, which implies some unusual changes in the observations in one quantity but not in the other.

**Figure 6:**
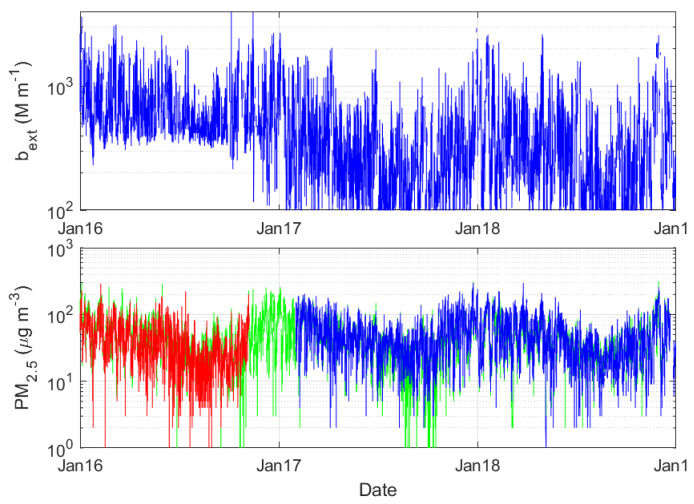
Time series of b_ext_ (**upper**) at Yangzhou, Jiangsu Province, and PM_2.5_ (**bottom**) in three adjacent stations.

It is interesting to note that PM_2.5_ observations at three adjacent stations (within 5 km) are close to each other and show a similar pattern in the three years of the study. Conversely, b_ext_ shows a striking phenomenon, that is, in 2016, it is extremely different from 2017 and 2018 (Figure 6). The relatively larger b_ext_ cannot be supported by contemporaneous PM_2.5_ observations. PM_2.5_ in these three years at these three stations shows a consistent and stable variation. The abnormally high b_ext_ in 2016 is very likely owing to the calibration or replacement of the visibility sensor, although this needs to be checked against the metadata.

**Figure 7:**
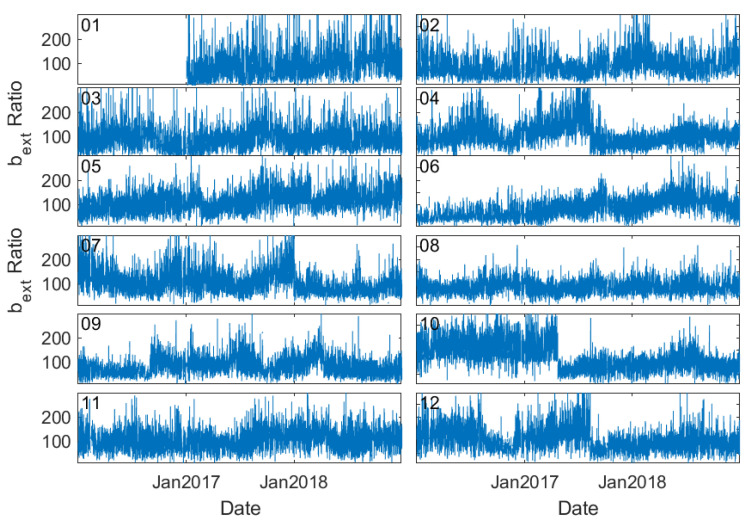
Time series of the ratio of b_ext_ at an individual station to the regional mean in Tianjin, a municipal city near Beijing.

**Figure 8:**
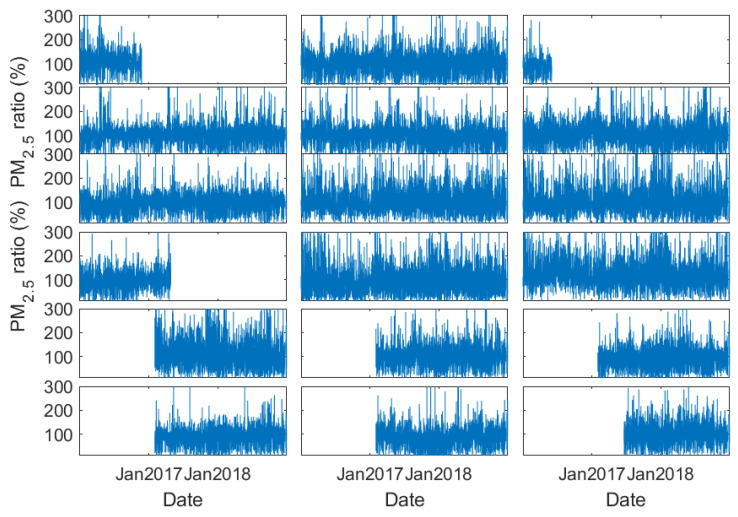
As Figure 7 but for PM_2.5_ data at 18 stations in Tianjin.

Abrupt changes in visibility and thereby b_ext_ are also evident from another perspective. Figure 7 presents the time series of the ratio of station b_ext_ to the regional mean b_ext_ in Tianjin, a municipal city near Beijing. A sudden drop in b_ext_ values occurred at stations 04, 07, 10, and 12 at the beginning of 2018, which cannot be reflected by PM_2.5_ measurements (Figure 8). Since the stations are located in a very small region, this abrupt and inconsistent change of b_ext_ is very likely associated with the recalibration or alternation of the sensors.

**Figure 9:**
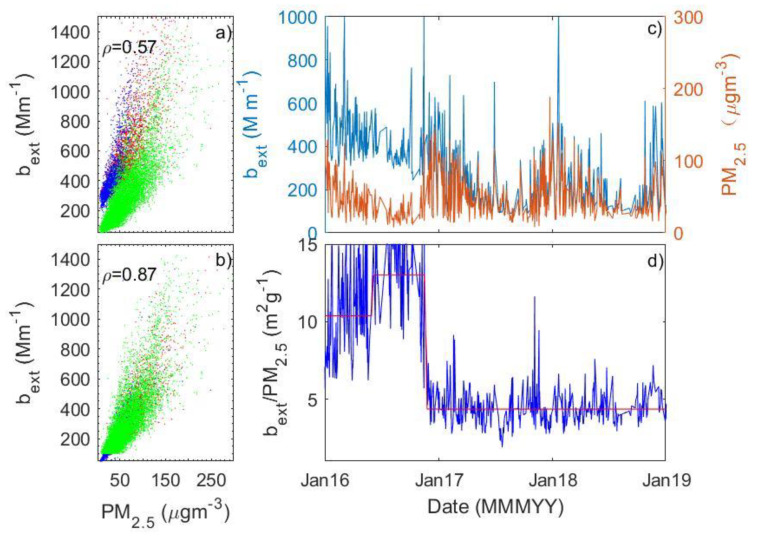
The relationship between PM_2.5_ and b_ext_ at Yangzhou, Jiangsu Province based on original and corrected measurements (**a**,**b**); the time series of PM_2.5_ and b_ext_ (**c**) and the ratio of b_ext_ to PM_2.5_, i.e., α_ext_ (**d**).

**Figure 10:**
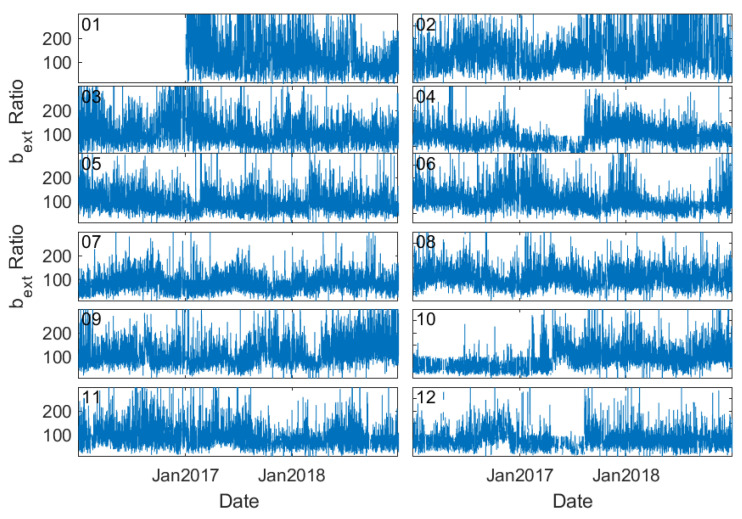
As Figure 7 but with b_ext_ corrected by using the method introduced in the text.

Given the fact that PM_2.5_ is stable but visibility, and hence b_ext_, drifts with time, the time series of α_ext_ should show abrupt change points. Therefore, a simple but effective method developed by Killick et al. [37] is used to detect those change points. This method can detect abrupt changes in the mean, variance, and trend of the time series; we only detect the change points of the mean values of α_ext_ here. Figure 9 presents an example of this analysis. The value of α_ext_, i.e., the ratio of b_ext_ to PM_2.5_ under RH < 40%, shows two change points (Figure 9d), which can be also clearly shown by the scatter plot of PM_2.5_ and b_ext_ (Figure 9a). Since α_ext_ values during the first two periods are abnormally higher than the expectation, indicating the abnormal measurement of b_ext_ (Figure 9b), b_ext_ measurements are then corrected by taking the measurements during the third period as the benchmark. Linear regression between b_ext_ and PM_2.5_ during the three periods is performed, leading to three linear equations. The questionable b_ext_ values during the first two periods are then corrected by using the following equations.

(4) $$b_{\mathrm{ext}}^{i}=b_{\mathrm{ext}}^{i}\cdot \frac{A_{r}+B_{r}\cdot {\mathrm{PM}}_{2.5}^{i}}{A_{i}+B_{i}\cdot {\mathrm{PM}}_{2.5}^{i}}$$

where i is the first or second period, and A_i_ and B_i_ represent the intercept and slope of the linear equation for the first or second period. The intercept and slope of the linear equation for the third period, the reference period, are represented by A_r_ and B_r_, respectively. The corrected result is shown in Figure 9b, which shows a much better correlation between b_ext_ and PM_2.5_ (0.87) than before (0.57) (Figure 9a). As shown in Figure 10, the time series of the ratio of corrected b_ext_ at an individual station to the regional mean in Tianjin are much more homogeneous compared to the series in Figure 7.

The method above is also suitable for the correction of visibility. Based on the method developed above, the visibility or b_ext_ data of the selected 502 stations are detected and corrected by comparison to the reference PM_2.5_ measurements, and, therefore, the visibility in these stations is set as the benchmark. In addition to the 502 collocated stations, the corrections of the 1893 remaining visibility stations should be related to nearby visibility measurements. If there is a benchmark station nearby (<30 km), the visibility adjustment in the target station refers to the benchmark station. Otherwise, the regional average visibility of all meteorological stations within 100 km in diameter is set to the reference series. After all corrections are completed, a dataset consisting of three years worth of visibility and b_ext_ data is finally produced.

## 4. Discussion and Conclusions

An analysis of the similarities and differences between visibility and PM_2.5_ measurements shows implications for the potential application of visibility observation to the study of air quality. Based on the quality-controlled PM_2.5_ and visibility data from 2016 to 2018, the nonparametric Spearman *ρ* value between two stations for PM_2.5_ and b_ext_ decreases as the distance between the stations, *R*, increases. The decay of *ρ* with *R* could be represented by an exponential equation. The relative uncertainty in PM_2.5_ measurements is 15% and 20% for b_ext_. Some relatively lower *ρ* values (< 0.4) observed for *R* < 50 km occur in regions characterized by the lowest (background) levels of PM_2.5_ and b_ext_ values, for example, the Tibetan and Yungui Plateau. The relatively smaller *ρ* for b_ext_ between stations than that of PM_2.5_ is probably caused by the predefined maximum threshold of visibility measurements (generally 30 km) and may be an obstacle to the application of visibility measurements in the study of air quality.

A significant correlation between PM_2.5_ and b_ext_ is derived in most stations. The percentages with significant correlation are 91%, 89%, 88%, and 93% from spring to winter. Relatively larger *ρ* values are evident in eastern China (Northeast China excluded) and in winter (the national median *ρ* is 0.67). A temporal variation in the relationship between PM_2.5_ and b_ext_ is evident in some stations, mainly caused by the abrupt changes in b_ext_ that are detected by an efficient approach developed by Killick et al. [37]. This implies a potentially large influence of alternation of visibility sensor instruments or re-calibrations. Therefore, the b_ext_ data are corrected by comparison to the reference measurements at the adjacent stations. A dataset of three years worth of visibility and b_ext_ data, which would be a complementary network to the study of air quality study, is finally produced.

To the best of our knowledge, this is the first time that a thorough check of the quality of automatic measurements of visibility in China has been made. Human observations of visibility in China are used by researchers to retrieve PM_2.5_ concentration, which shows the great potential of visibility measurements in the study of air quality. A great development in visibility measurements in China has been the use of visibility sensors to replace human observation. Instrument-based measurements of visibility is an objective rather than subjective measure and can provide measurements with high temporal resolution. However, we should keep in mind that instrumental measurements suffer a lot of issues that require a thorough evaluation of the obtained visibility data. Data quality is of high priority for the further application of these valuable data in air quality studies. We carefully evaluate the visibility measurements by collocating them with PM_2.5_ measurements in this study. It is clearly shown that visibility measurements are indeed associated with notable uncertainty. We develop a simple but effective to correct the visibility measurements. In particular, the systematic differences discussed above have been corrected at some stations. The quality of visibility data is greatly improved, which paves the way to use visibility data in air quality studies.

## Data Availability

All the datasets have been made available through the national meteorological services website (http://10.1.64.154/portal/web-home.index/, accessed on 1 November 2022).
